# Employment Models to Attract and Sustain Rural Generalist Doctors: Barriers and Enablers

**DOI:** 10.1111/ajr.70019

**Published:** 2025-03-14

**Authors:** Belinda O'Sullivan, Shane Boyer, Angela Stratton, Jacque Philips

**Affiliations:** ^1^ School of Rural Health Monash University Bendigo Victoria Australia; ^2^ Department of Health Victorian Rural Generalist Program Victoria Australia

**Keywords:** general practice, procedural, rural doctors, rural generalist (RG), Victoria

## Abstract

**Objective:**

Explore the barriers and enablers related to employment models to attract and sustain rural generalist (RG) doctors emerging from a state‐wide RG program.

**Setting:**

Stakeholders across five rural regions in Victoria, associated with the Victorian Rural Generalist Program (VRGP).

**Participants:**

36 stakeholders who responded from a list of 122 potential identified by a state‐wide advisory group for the VRGP. Respondents included 13 program staff, nine people from related collaborative agencies, four state policy decision‐makers and 10 RG employees/supervisors.

**Design:**

In‐depth interviews were thematically coded through ongoing reflection and sense‐making and verified by an independent qualitatively trained researcher.

**Results:**

There were four barriers to employment models to attract and retain RGs and three enablers. Barriers included health service competition, variability in health service leadership and executive capabilities/competencies regarding the RG workforce, specialist competition and supply‐based planning. Enablers were a state‐wide vision, improving regional‐level planning and RG recognition and reward.

**Conclusion:**

The research offers insights about the need for state‐wide, coordinated regional and local levels of strategy, planning and implementation to achieve employment models that attract and sustain RGs. Further, attracting and sustaining RGs depends on establishing and implementing clinical service models for the range of RGs needed in the community, using outpatient clinics and other networked service options across the region. It also relies on appropriate pay that recognises the additional skills that RGs have. The results might inform Australia's National Rural Generalist Pathway and the state and territory RG programs and their coordination units with progressing attractive and sustainable RG employment.

**Summary**

- What is already known on this subject?
  ○ Training pathways for doctors who work in both primary health care and other specialist areas in small rural communities (Rural Generalist or RG) are rapidly expanding in Australia.
  ○ But there is limited contemporary evidence identifying the barriers and enablers to employment models to attract and retain RG doctors who train in rural locations.
  ○ Available evidence hints that RGs rely on sufficient caseloads, cooperative clinical networks and supportive, engaged health services, for satisfactory employment.
- What this paper adds?
  ○ Evidence from diverse RG stakeholders identifies four barriers to RG employment in the context of Victoria's early implementation of the Victorian Rural Generalist Program (VRGP) which were health service competition, variability in health service leadership and their capability for RG workforce development, specialist competition and supply‐based planning.
  ○ There were three enablers which were state‐wide vision, regional‐level planning and recognition and reward.
  ○ The findings suggest that a systems approach is needed to enhance RG employment options.
  ○ The findings also provide insights for Australia's state and territory RG programmes as they progress with building attractive and sustainable RG employment, noting the importance of having a state‐wide vision for the RG workforce, coordinated regional planning, competency development for health service executives and reward and recognition for doctors who invest in RG training.

## 1 Introduction

Many small rural communities have limited access to specialist medical services and have restricted resources and the ability to travel for care elsewhere. They rely on access to locally based general practitioners (GPs) who have broader skills to cover a wider range of services in the community—a group which Australia refers to as Rural Generalists or RGs. RGs have been threatened by a wave of increasing medical specialisation [1] which is fuelled by the rewards of more narrowly focused careers [2], geographical narcissism [3] and technological advancements.

To build the RG workforce to equitably deliver services to rural and remote communities, many governments and rural regions worldwide are invested in RG‐focused training [4, 5, 6, 7]. This involves supervised vocational training centred around an RG curriculum, whilst doctors are based in smaller rural areas and developing connections which tend to impact their retention [8]. RG‐focused prevocational training also offers a bridge between medical school and RG vocational training in places like Australia [9, 10]. However, RG training opportunities can be localised in one region or state, with the most established models in Australia based in Queensland [8]. The Rural Doctors' Associations and rural stakeholders advocated for a more systematised approach to RG training and workforce development in Australia, resulting in the Australian Government investing in an Office of the National Rural Health Commissioner, which was tasked with developing a National Rural Generalist Pathway (NRGP) [11, 12]. The national pathway was informed through significant consultation, evidence and engagement, and final Taskforce Advice about the pathway was endorsed by the Health Minister in 2018 [12]. This flowed onto each jurisdiction (6 states and 2 territories in Australia) receiving a share of $62.2 million dollars in 2019 to implement Coordination Units and commence the development of integrated RG training pathways for RG workforce development [13].

The World Health Organisation's retention guidelines [14] and the WHO‐sponsored Rural Pathways Checklist [15] clearly delineate that retaining rural health workers relies on satisfactory working conditions for recruitment and retention. Unfortunately, if employment models are often not designed to attract and keep emerging doctors, then the investment in training is likely to be wasted. For the many stakeholders involved in RG training, rural towns, health services, staff and governments, supervisors and the doctors in training, this is an important issue because they invest a great deal into rural pathways using limited resources [16, 17]. Yet there is restricted research about the barriers and enablers of employment models to attract and retain RGs. What is available is somewhat outdated and mostly focuses on RGs working in procedural fields.

The available evidence suggests that maintaining RG procedural skills can be hindered by a lack of opportunity, the burden of multiple credentialing requirements across services where RGs work, and the time and expense that the RG incurs to maintain procedural skills [18]. RGs also face pressures related to the increasing medicalisation of childbirth and general emphasis on sub‐specialisation [19]. Potential solutions have been published, including improving access to assistance and backup for RGs through regionalised service networks of RGs and other specialists who support each other, inclusive of opportunities to continue up‐skilling in procedural skills [20]. Another study of early career procedural RGs identified that RGs may stop using their procedural skills over the first 5 years of practice (use dropping from 75%–61%); the first year post training was considered critical and 21% reported very poor support in that year [21]. GPs practising procedurally are more common in towns of 15,000 or less population, but the work involves around 10 h of additional labour in hospital settings, with no reduction in private practice workload [22].

Hospital decision‐making dynamics are also impactful on RG service sustainability, with research identifying that despite a service having good clinician‐led, patient‐centred care, a managed clinical network model failed because it did not engage fully with the regional health service management (unsupportive macrosystem) [23]. Other networked rural maternity services have also struggled due to staffing challenges requiring highly adaptable models to cope with local demand [24]. Robinson et al. also noted the gradual decline in the volume and complexity of procedural work in rural areas underpinned by changing community demographics, service closures and rising specialisation, leading to centralised services [25]. Although employment to attract and sustain the RG workforce is complex, it is critical to understand because procedural work is associated with improved rural retention of GPs [26].

With this background in mind, this study aimed to explore the barriers and enablers related to employment models to attract and sustain RG doctors emerging from the VRGP in Victoria, which is one of the many state‐wide RG training programmes which was implement as part of Australia's National Rural Generalist Pathway.

## 2 Methods

The project was ethically approved via Monash University (ID#30309, 6 September 2021) and it complied with ethics and privacy protocols.

### 2.1 Conceptual and Political Frame

This study was based in Victoria (a state of Australia) which already had RG‐focused internships since 2012 and since 2019, had used funding from the National Rural Generalist Program to set up a Coordination Unit to build a longitudinal 6 year pathway based on this (year 1 and 2 cohorts had been recruited at the time of this study in 2021). The first year of the VRGP was the Rural Community Internship Programs which were in place in all rural regions at the time [9]. Victoria had no state award for RG employment. Victoria defined RG doctors in line with the national definition [4], as qualified GPs with emergency skills and additional speciality‐level qualifications, who work in hospital and/or community settings as part of a rural healthcare team [27]. The Victorian health services are fully devolved from the State Health Department and led by local boards of governance, such that decision‐making by each health service is independent from others (Box 1).

BOX 1 **Victorian health system structure and governance.**

Victoria uses devolved health service governance, and its geographic and population distribution is centred on five major regional centres, which is a unique context for building the RG workforce. Victoria has 300 hospitals and health services, including public and private hospitals across its five regional catchments, along with other smaller independent rural health services [28]. Predominantly, these are governed by independent boards of directors who make local decisions to meet local needs premised on a model of responsive regulation, that is, accountability through agreed mechanisms or intervention where self‐regulation fails [29]. This means that action is expected to be taken by the board to assure that health services are safe and high quality, legally compliant, accessible, efficient, and financially viable [29]. The Victorian Department of Health plays a role in setting policy but mainly relates to the health services through individual contractual arrangements with each service.

The study was commissioned by the Victorian Department of Health. The department was motivated to understand how to optimise RG training and employment in Victoria to promote the success of the VRGP. The background to RG employment models is provided in Box 2.

BOX 2 **RG employment models.**

The predominant RG employment model in Victoria encompasses doctors employed in a private general practice or Aboriginal Community Controlled Health Organisation (ACCHOs), who attend hospitals as a Visiting Medical Officers (VMO) [27]. This is similar in other states; however, RGs in Victoria negotiate their employment terms and conditions with independent health services, rather than local health networks (described in Box 1).

In Queensland, where there are local health networks, RG medicine was formally recognised through a certified agreement in 2008. This came with salary classification for Senior Medical Officers to provide remuneration equivalent to RGs working at the scope of other specialists in the hospital system [30]. Further reform provided a similar value for Queensland's Visiting Medical Officer (VMO) model [30].

Before commencing the research, the research team met with the department to gain a broad understanding of the VRGP design, also reading program documentation and regularly meeting with a VRGP state‐wide advisory group that was established for this project.

The research used a grounded theory approach and systems thinking to deduce patterns from the data that will inform a complex adaptive rural training and employment models, without pre‐conceived notions of hypotheses [31].

### 2.2 Sampling Frame

Stakeholder interviews were the method of choice to answer the research question. A list of 122 potential interviewees was developed in consultation with the VRGP state‐wide advisory group and aligned with purposive, maximum diversity sampling [32]. The list included VRGP‐knowledgeable people from the Victorian Department of Health, the State‐wide advisory group, VRGP Regional Networks, VRGP staff, the Postgraduate Medical Council of Victoria and Rural Community Internship Programs (1st year of VRGP training), health services employing RGs and RG supervisors.

### 2.3 Procedure

All 122 stakeholders were contacted by the lead researcher by email between August and September 2021, provided an overview of the study and a link to the information about the study. Those interested consented and enrolled. There was no financial reward. The interviews were of 1 h duration and occurred through video‐consultation software between September and October 2021. The interviews were semi‐structured, piloted with the advisory group and refined by the researcher over time to explore emerging phenomena.

The interviewer was a rural health academic who was independently employed for this project Some participants were loosley known to the interviewer through the rural networks she operated in.

The interviewer recorded, transcribed, and reviewed transcripts for accuracy. Further, following each interview, the interviewer took reflective notes to aid interpretation and help to identify relevant topics to expand understanding of in subsequent interviews. The interviewer also engaged in regular meetings with the research team and the state‐wide advisory group to shape understanding of the data.

### 2.4 Analysis

The emerging data representing nearly 40 h of verbatim evidence. Data were anonymised to service, town and aggregated by respondent type including program staff, staff from collaborative agencies, state policy decision‐makers and employees/supervisors, denoted through sub‐text ‘p’, ‘c’, ‘s’ and ‘e’ respectively to aid the interpretation of quoted material.

The transcripts were read and re‐read by the research team for sense‐making (consisting of VRGP staff and employers, one of whom was an RG doctor and all had experience of RG training). Codes were progressively shaped and discussed to inform key themes, which were given time to mature through regular meetings of the research team between October 2021 and March 2022. The Braun et al. method [33] was used. The first read‐through was done in detail and an initial set of codes was applied. These codes were re‐visited as transcripts were re‐read to build up more consistent themes through inductive analyses, regular reflection and re‐arranging of the data [33]. This process continued until a set of agreed final themes emerged that were considered to represent the data [33] and saturation was achieved. The themes were presented regularly to the research team and later to the wider state‐wide advisory group for clarification, and this informed interpretation and was done to reduce subjective bias [34].

The final themes were then independently reviewed by a separate qualitatively trained researcher who was contracted for this work. This researcher was not familiar with RG training but was briefed about the material, and she confirmed the themes were accurate.

## 3 Results

Overall, 36 stakeholders responded (30% response rate) and consistent with maximum variation sampling respondents included 13 program staff, 9 respondents from collaborative agencies, 4 state‐level policymakers and 10 employees/supervisors. The spread of respondents included 14 males and 22 females, spread equally across Victoria's five rural regions. More than half (53%; *n* = 19/36) had two or more years' experience of RG training. Seven key themes emerged encompassing four barriers and three enablers, and there were no minor themes.

### 3.1 Barriers

#### 3.1.1 Competing Goals Between Health Services

Within the context of Victoria's devolved health service governance model where all services are independent of each other, competition between services impacted RG employment opportunities. Smaller services were viewed as deficient by larger ones, and workforce resources were withheld or not shared as expected.

All the small places think the large places are bullies, and the big places think the small places are inadequate, there is fault everywhere. (e7)

The larger health services did not hold RG workforce as a priority so it was hard to develop regional thinking about RG workforce.

RG is a small issue for the big health services, they don't mean anything to them…. (p18)

#### 3.1.2 Variability in Leadership Capability & Focus

The turnover and varying competencies of health service executives concerning the RG workforce (including their understanding of RG work models over various procedural and non‐procedural components, in different services) was a barrier to engagement over building suitable RG employment models.

…varying abilities of the CEOs in health services to understand the medical workforce structure. It's complex and it take years to understand… then there are good ones… they move, so the health service is a house of cards… (e27)

Further, person‐dependencies and self‐interests were evident.

[decisions]…can be personality based… (e27)

#### 3.1.3 Specialist Competition

The non‐RG specialists competed with RGs over employment options, with evidence of self‐interest.

The specialists who don't believe it get high positions and set up the turf, they protect their space… (p8)

And medical power of the specialists trumped the capacity of health service executives to engage with the RG model.

…the smaller health services, they need to play with specialists, or their service will be withdrawn. They blackmail … “if you have an RG, we will not deliver any surgery” …(p16)

#### 3.1.4 Supply‐Based Planning

There was a sense of employment models being planned around available staff, rather than how to meet community needs in the most cost‐effective way, partly fuelled by a sense of an overall workforce crisis in rural areas.

…small rural health services are still designing their models around whatever doctor they can get. (e31)

RG employment models were built sporadically around the interests and skills of doctors already in the community, rather than by planning training and employment models to fit the needs of the community.

I need to talk with … other CEOs and brainstorm how to keep the RG with emergency skills in our community. (e32)

### 3.2 Enablers

#### 3.2.1 State‐Wide Vision

It was viewed that setting a state‐wide vision for the RG workforce could improve clarity and assist with the planning of RG employment models that were attractive and able to sustain RGs.

The Department of Health needs to … lead and be clear where we need an RG … make it clear about what models are useful. (p22)

Understanding the employment model was seen as a pre‐requisite to designing the sort of training that RGs needed to get.

… we need to know the employment model and how the training fits it. (e12)

#### 3.2.2 Regional‐Level Planning

Responses highlighted that regional‐level planning of health service models to meet community needs, along with a roadmap for coordinated delivery, would assist health services within the five regions to work together for RG employment models.

… the health services to come together and plan…a clear forward plan on the needs and how we will deliver? (e27)

This was considered to rely on a unified understanding of RG credentials and accreditation requirements beyond the current arrangement where doctors individually negotiated with each health service.

[onus on] an individual doctor to negotiate credentialing for any role. (c15)

Good regional planning requires consideration of employment models that enable RGs to work with sufficient caseloads and provide safe and high‐quality services in the location.

Is the workload safe enough to keep you trained as an RG in that location. Or should there be a local health network for an anaesthetist to be shared around? (p13)

#### 3.2.3 Recognition and Reward

It was noted that in Victoria, RGs earn no additional income compared with non‐RG trained doctors and less than non‐GP specialists for providing the same level of service. However, an enabler was improving the level of recognition and reward for RGs who train through substantive appointments in hospitals.

The job at the end [of the training pathway should be], a substantive appointment…outpatient clinics in mental health …and acute and chronic components. (e31)

Moreover, through more billings for the RG's additional specialist skills, not just in procedural areas where credentialing is currently clearer, but in areas like paediatrics, palliative care and other clinics based in the community.

Anaesthetics, emergency, and obstetrics are the standout for credentialing. You bill the same for the other non‐procedural things, so what is the appeal of doing that? (p18)

This was considered to rely on improvements to Australia's Medicare Benefits payment systems to ensure:

the RGs are…valued. (p22)

## 4 Discussion

This study provides some important findings to build employment which improves the attraction and retention of RGs emerging from RG training, whilst acknowledging the unique health service context, geography, and medical workforce dynamics of Victoria. The findings suggest that attractive, sustainable RG models can be enhanced by addressing key barriers and enablers. The implications are summarised in two key areas.

Firstly, a key solution to promote attractive, sustainable employment is to develop a state‐wide vision statement, methods for coordinated regional‐level planning and implement state‐wide credentialing frameworks.

The research suggested that the vision of what a ‘good’ RG workforce looks like in Victoria was relatively under‐developed, although it needs to cover both procedural and non‐procedural areas. A high‐level framework of relevant employment options for services to apply could assist uptake. This vision must be clear about how specialist hubs in bigger regional centres and their outreach services [35] and other online service models, might overlay and support an attractive and sustainable RG workforce model. Orientation, training and regular up‐skilling about the RG workforce, along with a state‐wide credentialing framework, might help to build health service executive capability for adopting and trusting RG service models. Regionally networked strategic planning might also assist in sourcing and managing RG caseloads with appropriate backup from other specialists. This sort of approach might be a better planning model to build health services to meet community need in the most cost‐effective way, rather than playing into competition between individual services and RGs competing with medical specialists. Given the size of rural workforce shortages in many states and territories, supply‐based planning is a reality in most rural regions; however, the findings suggest this should not occur at the expense of forward planning because junior doctors and registrars involved in RG training may not be retained unless there are satisfactory and sustainable RG jobs at the end [2].

Encouraging regional level planning for RG workforce is one of the roles of the VRGP Coordination Unit. They host Regional Network meetings in each region which focus on RG training and workforce planning. However, participation currently relies on the level of interest and engagement of various stakeholders in the network. Victoria recently formed Health Service Partnerships to promote networking of independent hospitals to progress strategic system priorities, and there aer wider health service amalgamations underway in Victoria which could be a foundation for unified governance and strategic planning of RG employment models going forward [36].

**FIGURE 1:**
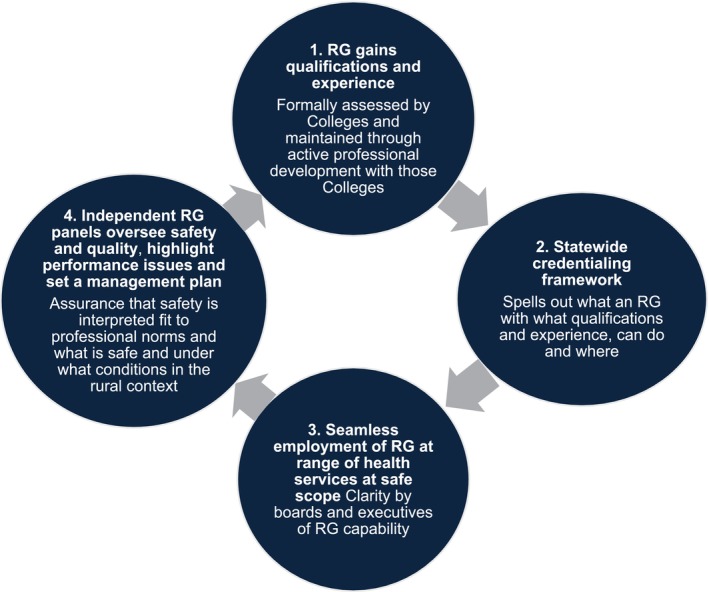
Pathway to seamless employment models.

Currently, however, Victoria manages the credentialing of doctors through a committee at each health service, normally convened by the Director of Medical Services [37]. Credentialing can be undertaken at the local, subregional, regional or state level. Health services are encouraged to facilitate a shared credentialing and review process, but approval is undertaken at the individual health service level and needs to align with the specific roles in each health service. A state‐wide RG credentialing model could standardise understanding and improve the implementation of coordinated RG employment across the state, enabling better endorsement of safe and high‐quality RG‐led services. The Taskforce Advice on the National Rural Generalist Pathway suggests that such Frameworks should be informed by RG peer review to ensure that they operate with insights into the nature of safe practice in small rural contexts where RGs work (Figure 1) [12]:

Secondly, the research suggests that employment to attract and sustain RGs in Victoria depends on developing substantive RG roles in rural hospital/outpatient clinics with specialist‐level pay for their additional skills areas.

**FIGURE 2:**
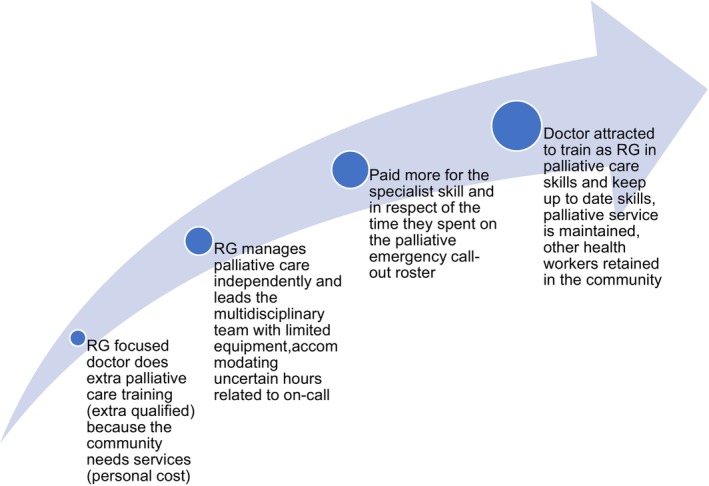
An example of recognition and reward model for RG focused in general practice and palliative care.

Recognition and reward needs to follow different procedural and non‐procedural specialist skills along with general practice work (where rural communities mostly rely on the latter for primary and preventative healthcare). For an RG to pursue the extra training for the additional specialist skills, they need appropriate additional remuneration for those skills (Figure 2). This aligns with national and international evidence identifying that rural recruitment and retention relies on health workers being rewarded for the training they have done in extra qualifications [12, 14]. Wider theory about choosing a generalist‐focused career highlights that medical students and junior doctors choose generalist careers if they can see sustainable jobs where they can put all of their skills together [2]. The co‐investment of the Australian and state governments in developing single‐employer model trials (for salaried hospital and community employment of RG registrars) is a lever to use for this purpose; however, they may need to be scaled up to align with the footprint of the National Rural Generalist Pathway and need to be sustained beyond the training years if the intention is to retain RGs [38]. Given the complexity of RG work, achieving the right employment package may require multiple health services and general practices to work together across a region for access to interesting/valid caseloads for sustainable and satisfying RG practice. This is supported by the findings of other literature about RGs working in obstetrics and other procedural fields [18]. Regionalised models and single‐employer models also make sense in non‐procedural fields of practice, but they have had less focus within policy and research.

Financially rewarding health workers who train and work in rural careers is known to be an important issue for achieving a workforce retention, based on WHO guidelines [14]. In Queensland (another state of Australia), RG medicine was formally recognised through a certified agreement in 2008 along with salary classification for Senior Medical Officers to provide remuneration equivalent to RGs working at the scope of other specialists in the hospital system [30]. Nationally, the GP colleges have lodged an application with the Professional Regulator to approve RG as a specialty within the field of General Practice. If approved, it might stimulate further action at the state level, related to developing State awards and improving national Medicare rebates (contributing to community‐level billings when not working on salary), to remunerate additional skills for those who are RG qualified. These options seem particularly critical given the next generation of doctors expresses an interest in working in more salaried part‐ or full‐time employment for security and work‐life balance [39, 40].

This study had limitations. It was only based on qualitative data which did not identify where RGs are currently working or for how long. It is also limited to Victoria, which has unique geography and service arrangements, at the early stages of implementing RG training. Due to the diversity of stakeholders interviewed and the complexity of the topic, it is possible that there are other themes that were not identified. The current study did not interview RG trainees, which means that their perspectives were not evident in the findings. The latter group could have identified other barriers and enablers. Some insights from RGs are incorporated; however, because a range of participants were clinically active RGs associated with the program in some capacity.

## 5 Conclusion

This study provides some insights as to the nature of the barriers and enablers to employment models to attract and retain RGs in a state where RG training is emerging. Four barriers and three enablers suggest that improving the situation may require a systems approach, including setting a clearly understood vision for RG employment to meet community needs and ensuring this articulates with coordinated regional‐level planning across regional health services. Health service executives could be better supported by orientation, training and up‐skilling about the RG workforce. Implementing a state‐wide credentialing framework for RGs could assist decision‐making around employment. Further, specific substantive roles in health services and recognition and remuneration for hospital and community specialist services are likely to attract and sustain RGs. With clear vision, planning, recognition and financial reward, there is the potential for improved employment to attract and retain RGs emerging from RG training programs.

## Author Contributions

**Belinda O'Sullivan:** conceptualization, methodology, data curation, validation, supervision, visualization, resources, writing – review and editing, writing – original draft, project administration, funding acquisition, formal analysis, investigation. **Shane Boyer:** conceptualization, methodology, validation, writing – review and editing. **Angela Stratton:** writing – review and editing, validation, methodology, conceptualization. **Jacque Philips:** conceptualization, methodology, validation, writing – review and editing.

## Disclosure

This research is included in an internal report about the VRGP evaluation, which was submitted to the Victorian Department of Health (not for public release) and is available with its permission.

## Ethics Statement

The project was ethically approved by Monash University (ID # 30309, 6 September 2021).

## Conflicts of Interest

The authors acknowledge this research was funded by the Victorian Department of Health, and all authors were employees of the Department for the duration of this project. The main author was employed as an independent consultant for this research, and these potential conflicts were clear to the participants of this project.

## Data Availability

The data that support the findings of this study are available on request from the corresponding author in consultation with the Victorian Department of Health. The data are not publicly available due to privacy or ethical restrictions.
